# Governing Antimicrobial Resistance (AMR) in a Changing Climate: A Participatory Scenario Planning Approach Applied to Sweden in 2050

**DOI:** 10.3389/fpubh.2022.831097

**Published:** 2022-07-06

**Authors:** Irene Anna Lambraki, Melanie Cousins, Tiscar Graells, Anaïs Léger, Sara Abdelrahman, Andrew P. Desbois, Rose Gallagher, Birgitta Staaf Larsson, Bengt Mattson, Patrik Henriksson, Max Troell, Peter Søgaard Jørgensen, Didier Wernli, Carolee Anne Carson, Elizabeth Jane Parmley, Shannon Elizabeth Majowicz

**Affiliations:** ^1^School of Public Health Sciences, University of Waterloo, Waterloo, ON, Canada; ^2^Global Economic Dynamics and the Biosphere, Royal Swedish Academy of Sciences, Stockholm, Sweden; ^3^Stockholm Resilience Centre, Stockholm University, Stockholm, Sweden; ^4^Global Studies Institute, University of Geneva, Geneva, Switzerland; ^5^Faculty of Natural Sciences, Institute of Aquaculture, University of Stirling, Stirling, United Kingdom; ^6^Royal College of Nursing, London, United Kingdom; ^7^Swedish Centre for Animal Welfare, Swedish University of Agricultural Sciences, Uppsala, Sweden; ^8^LIF, The Swedish Pharmaceutical Industry Association, Stockholm, Sweden; ^9^Beijer Institute of Ecological Economics, Royal Swedish Academy of Sciences, Stockholm, Sweden; ^10^WorldFish, Penang, Malaysia; ^11^Centre for Food-Borne, Environmental and Zoonotic Infectious Diseases, Public Health Agency of Canada, Guelph, ON, Canada; ^12^Department of Population Medicine, Ontario Veterinary College, University of Guelph, Guelph, ON, Canada

**Keywords:** antimicrobial resistance (AMR), climate change, Sweden, scenario planning, interventions, Sustainable Development Goals (SDGs), alternative futures

## Abstract

**Background:**

Antimicrobial resistance (AMR) is a growing global crisis with long-term and unpredictable health, social and economic impacts, with which climate change is likely to interact. Understanding how to govern AMR amidst evolving climatic changes is critical. Scenario planning offers a suitable approach. By envisioning alternative futures, stakeholders more effectively can identify consequences, anticipate problems, and better determine how to intervene. This study explored future worlds and actions that may successfully address AMR in a changing climate in a high-income country, using Sweden as the case.

**Methods:**

We conducted online scenario-building workshops and interviews with eight experts who explored: (1) how promising interventions (*taxation of antimicrobials at point of sale*, and *infection prevention measures*) could each combat AMR in 2050 in Sweden given our changing climate; and (2) actions to take starting in 2030 to ensure success in 2050. Transcripts were thematically analyzed to produce a narrative of participant validated alternative futures.

**Results:**

Recognizing AMR to be a global problem requiring global solutions, participants looked beyond Sweden to construct three alternative futures: (1) “Tax Burn Out” revealed *taxation of antimicrobials* as a low-impact intervention that creates inequities and thus would fail to address AMR without other interventions, such as infection prevention measures. (2) “Addressing the Basics” identified *infection prevention measures* as highly impactful at containing AMR in 2050 because they would contribute to achieving the Sustainable Development Goals (SDGs), which would be essential to tackling inequities underpinning AMR and climate change, and help to stabilize climate-induced mass migration and conflicts; and (3) ”Siloed Nations” described a movement toward nationalism and protectionism that would derail the “Addressing the Basics” scenario, threatening health and wellbeing of all. Several urgent actions were identified to combat AMR long-term regardless which future un-folds, such as global collaboration, and a holistic approach where AMR and climate change are addressed as interlinked issues.

**Conclusion:**

Our participatory scenario planning approach enabled participants from different sectors to create shared future visions and identify urgent actions to take that hinge on global collaboration, addressing AMR and climate change together, and achieving the SDGs to combat AMR under a changing climate.

## Introduction

Antimicrobial resistance (AMR) is a growing global crisis that claims approximately 700,000 lives per year (1) and may unleash severe and unpredictable negative health and economic consequences, with healthcare costs estimated at $300 billion to $1 trillion per year by 2050 (2). AMR occurs when infectious microorganisms survive in the presence of antimicrobials that are designed to control or kill them, and thus lead to weakened drug effectiveness. Antimicrobials, such as antibiotics, are relied upon in human and veterinary medicine and agricultural and aquacultural systems to treat illness, reduce deaths, and produce food animals efficiently and profitably (3–5); paradoxically, our use of antimicrobials is a major driver of AMR (6). Run-off from animal farms, pharmaceutical industries, and hospitals; waste and wastewater treatment plants; and international travel are believed to be key routes for antimicrobial dispersal and resistant bacteria and resistance gene transmission across human, animal and environmental systems (7–10).

AMR is a top 10 global public health threat (11), and while 117 countries worldwide have government-approved AMR National Action Plans (12) that align with the Global Action Plan for AMR (13), concomitant global challenges may threaten current AMR mitigation efforts. Evolving climatic changes is one such challenge. Human activities such as the burning of fossil fuels have led to the emission of greenhouse gases that is increasing the Earth's temperature (14). Increasing global mean temperature will bring heat waves and, if left unaddressed, temperatures could reach critical tolerance thresholds for agriculture and human health (15). Climate change will also cause increased frequency of catastrophic storms, intense rainfall and associated flooding and drought in different regions of our planet (15). Moreover, the proportion of land used for important activities, such as agriculture, will increasingly be negatively impacted in some areas (16). Climatic changes will also impact the occurrence of AMR and the problems associated with this phenomenon. For instance, as the Earth's temperature rises, physiological stress in animals will increase and lead to greater incidences of infectious diseases requiring antimicrobial use (AMU) (17). This is problematic because microorganisms adapt to temperature changes in the environment and with increasing temperatures are better able to pass on resistance to other microorganisms via horizontal gene transfer mechanisms that in turn will increase the challenge of treating infectious diseases with antimicrobials (18–20). Climatic changes such as flooding will drive human migration and increase overcrowding of people in particular areas, amplifying AMR transmission particularly in areas with poor sanitation infrastructure (20). Animals and humans will come into closer and more frequent contact, thereby increasing the potential for vector-borne and zoonotic disease outbreaks with pandemic potential (20). Severe weather events will threaten food security that may cause people to consume contaminated foods leading to foodborne illnesses requiring AMU treatment (21). Low- and middle-income countries and people of lower socio-economic status will more likely experience the disproportionate burden of climate change and AMR, making these problems social justice issues (20, 22). Addressing AMR effectively and sustainably therefore requires planning with the future in mind. Exploring which future might un-fold is important to anticipate possible risks, test whether existing strategies and plans will address them, and determine other actions that may be required to deal with the uncertainties that arise.

Scenario planning is a type of foresight method that enables exploration of alternative future worlds that might come to pass, and this approach helps to develop understanding about how those worlds can be realized or avoided (23). Shell Oil used scenario planning decades ago as a tool for strategizing future needs in energy supply and demand (24), and it has since been applied to e.g., the future of academic medicine (25), veterinary medicine (26), different agricultural and environment issues (27), and it has been identified as important to address complex public health problems (28). Unlike structured foresight methods such as forecasting that use existing data and predictive models to make predictions, scenario planning is often used when data and models are sparse or when numerous unpredictable factors influence an outcome, as is the case with AMR. Thus, scenario planning is often qualitative, narrative-based and benefits from participatory approaches that engage participants to construct alternative futures by exploring “what if” questions or pre-determined situations and then “back-cast” or work backwards to think through effective strategies to achieve or avoid future worlds (23).

Given the intersectoral nature of AMR, engaging multisectoral stakeholders in scenario planning exercises is necessary to construct alternative futures that are informed by diverse thinking about the broad forces that may impact the problem, and that allow us to explore each future's consequences to better identify risks and how to mitigate them. To our knowledge, such an exploration has not been undertaken previously for the problem of AMR under changing climatic conditions.

### Study Objectives

The aim of this study was to explore alternative futures and actions needed to successfully address AMR under a changing climate in the year 2050, using Sweden as the case. Sweden has demonstrated success in reducing AMR levels (29) while contending with importation of animal foods raised with variable AMU. Sweden is also embedded within Europe as a member of the European Union and is thus influenced by this broader context when introducing strategies, such as the European Union Green Deal (30) and Farm to Fork Strategy (31) that include AMU reduction goals and strive to make Europe carbon neutral by 2050, thereby providing a rich setting for exploring how to sustainably mitigate AMR under changing climatic conditions.

## Materials and Methods

This qualitative study addressed the Consolidated Criteria for Reporting Qualitative Research (COREQ) checklist for reporting qualitative research (32). The study received ethics clearance from a University of Waterloo Research Ethics Committee (ORE#: 41781). Participants provided written informed consent to participate.

### Research Team and Reflexivity

The study was designed, conducted, and analyzed by a core team (SMJ, EJP, CAC, IAL, MC, SA) who consulted with an international research team throughout the process. The disciplinary background of the core team comprised public health and epidemiology, with specializations in food safety, AMR, One Health (33), health promotion and qualitative research methods, and systems thinking. The international team had specialties relevant to human and animal health and medicine, clinical microbiology, and evolutionary biology. The international team was from Sweden and Europe while the core team was external to Europe, which enabled consideration of context relevant to the Sweden and broader international context throughout the study process.

### Study Design

**Figure 1:**
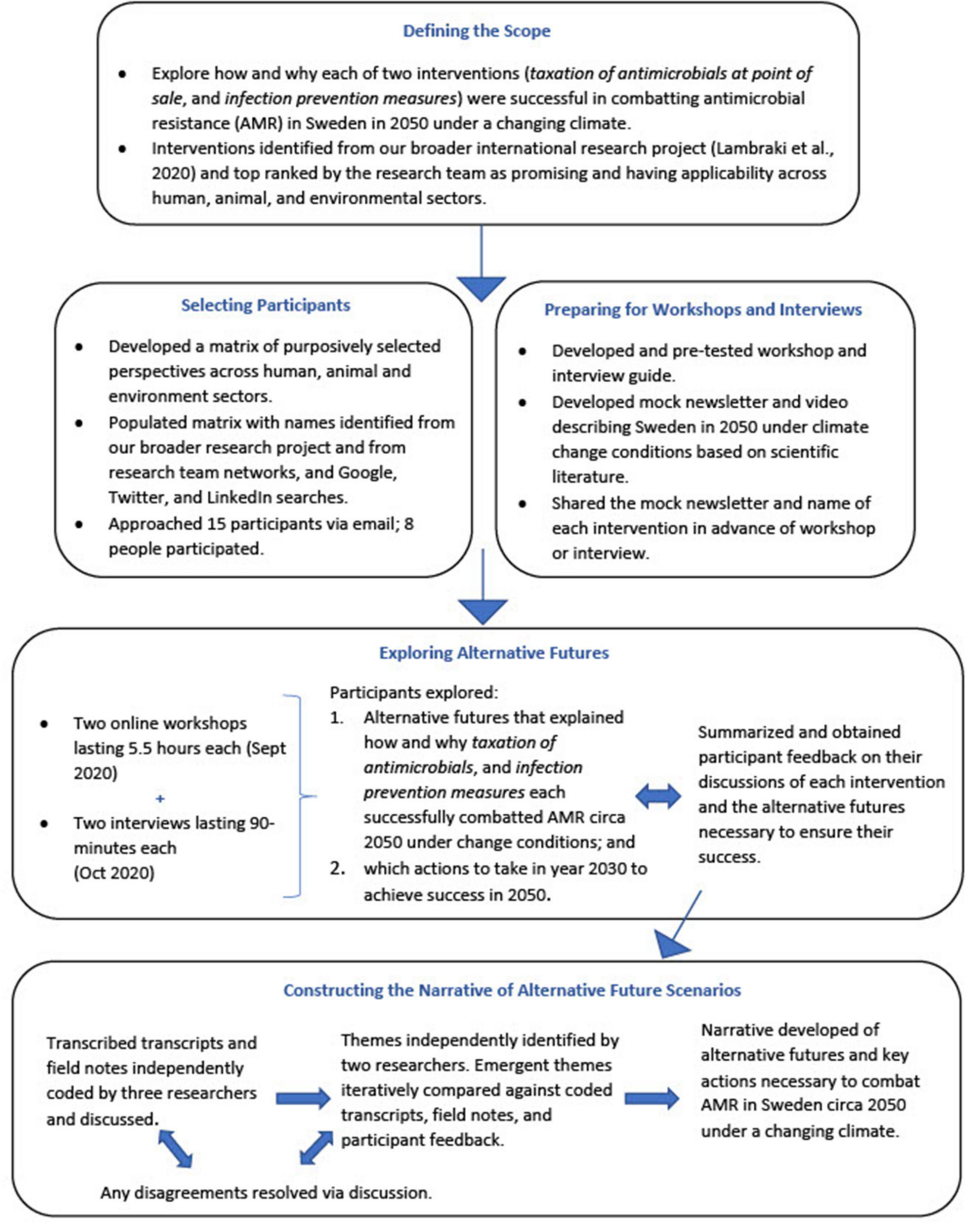
Scenario planning procedures.

We conducted a participatory scenario planning exercise that involved presenting diverse stakeholders with two pre-determined and desirable futures where AMR was successfully addressed under a changing climate circa 2050 in Sweden as a result of two promising interventions, then asking stakeholders to envision what the year 2050 would need to look like to ensure this success, and then “back-cast” (23) from these futures to determine what actions are needed to address AMR regardless what the future holds. Our approach involved a four-step approach: (1) Defining the scope; (2) Selecting Participants; (3) Exploring Alternative Futures; and (4) Constructing the Narrative of Alternative Future Scenarios (Figure 1).

#### Defining the Scope

Our research team convened to agree on the purpose and timeframe of our scenario planning approach, and focused on exploring how and why two interventions—*taxation of antimicrobials at point of sale* and *infection prevention measures*—could each effectively address AMR under a changing climate in Sweden in the year 2050. We focused on these two interventions because they would provide a point of discussion for participants to determine how Sweden would need to look in 2050 to ensure each intervention was successful considering climate change impacts. The selected interventions were identified from activities of our broader international research project (34), and top ranked by our team as promising interventions with application across the One Health spectrum.

#### Selecting Participants

We approached 15 participants based in Europe via email, purposively selected because they represented perspectives from different sectors across the One Health spectrum that may influence AMR under a changing climate. To select participants, our team developed a matrix of desired perspectives and populated it with individuals identified through a pool of candidates from our broader research project that had granted permission to be re-contacted (35), as well as new ones identified through: authors' professional networks; Google, LinkedIn and Twitter searches; and searches of professional organizations' websites. Participants that had been involved in our previous study had basic familiarity with some of the core research team members (IAL, EJP, MC). All participants were informed that they were not obligated to participate and could withdraw their participation at any time. In total, eight participants representing economics and trade, nursing, animal welfare, food safety and microbiology, aquatic sciences, agricultural crops and policy, pharmaceutical marketing and research, and urban and sustainable agricultural innovation took part in the present study. The remaining seven participants either did not respond or originally agreed to participate but subsequently declined due to COVID-19 issues or work-related conflicts.

#### Exploring Alternative Futures

Due to the COVID-19 pandemic, we conducted workshops online instead of in-person, using the Microsoft Teams platform. Workshops occurred on September 10th and 11th 2020 and lasted 5.5 h each day. Two interviews lasting 90 min each were conducted with two participants who were unable to attend one or both workshop days.

Workshops and interviews were audio-recorded, guided by a pre-tested semi-structured interview guide, and facilitated by our team (IAL and EJP as co-facilitators; MC as note taker). Workshop and interview procedures involved a review of the agenda, introductions, and a simple exercise to catapult participants into the year 2050. We then tasked participants to explore (1) how and why *taxation of antimicrobials at point of sale* and *infection prevention measures* were each successful in addressing AMR in Sweden in the year 2050 under a changing climate, and (2) which actions will need to be taken starting in the year 2030 to achieve success in 2050.

To assist discussions, participants were formally introduced to materials that they were sent three days in advance of the workshops or interview. Participants received a causal loop diagram of drivers of AMR in Europe [with a Swedish focus; (35)] to use as a tool for exploring potential intervention impacts on AMR if desired, as well as a mock news story (36) and accompanying video (37) describing Sweden under anticipated climate change conditions in the year 2050, including impacts on vector-borne diseases, infectious diseases, physical injuries, the food system and economics, and immigration and travel. The aim of the news story and video was not to accurately describe climate change projections, but rather to push participants into a possible climate change future and stimulate their thinking about how each selected intervention could be successful in Sweden circa 2050 and identify trends (e.g., continuing growth of environmental interest groups) and uncertainties (e.g., technological advancements) that may enable or derail success. To develop the storyline, evidence-based statements about climate change in Sweden and Europe were extracted from the literature and tailored to the Swedish context. Each of the two interventions was explored separately. We purposively kept descriptions about each intervention vague, allowing participants to determine details about them (e.g., whether the intervention would be applied to human, animal, or other settings) and to construct what the year 2050 would need to look like to ensure intervention success under a changing climate, and the actions needed starting in 2030 to achieve success. Participants were encouraged to define and make explicit the boundaries of their discussions, draw on their expertise, and to think creatively. Workshop and interview discussions continued until participants had no new information to share and indicated that their discussions were complete.

#### Constructing the Narrative of Alternative Future Scenarios

After all workshops were complete, transcribed verbatim transcripts, meeting notes, and field notes were then coded and analyzed thematically. Coding was carried out independently by three researchers (co-authors: SA, IAL; project coordinator: Jenna Dixon) and themes were identified and compared for consistency across two researchers (SA, IAL). Any differences in coding or themes were resolved through discussion. Prior to coding and thematic analysis, IAL created a summary of the 2050 alternative futures that participants had described by reviewing the audio-recorded sessions and detailed meeting notes and field notes, sharing these with participants for feedback to ensure they accurately reflected their understanding of discussions. Then, through an iterative process of triangulating emergent themes against transcripts, meeting and field notes, discussions with team members, and participant feedback, a narrative was developed describing participants' constructions of alternative futures where each intervention explored could be successful in addressing AMR in Sweden circa 2050 under a changing climate and the key actions necessary to take starting in the year 2030 to ensure success. Illustrative quotations (identified by workshop or interview day as per approved University of Waterloo research ethics requirements) are provided in the Results section.

## Results

To develop alternative futures, participants defined the boundaries of their discussions. First, participants determined to focus discussions both on Sweden and at the international level because participants recognized AMR to be a global problem that requires global solutions, noting “*there is no point of somebody doing something in one part of the world and somebody else not following in a different country or continent*” (Day 1 workshop). Second, participants determined to focus discussions on antibiotics and broadly on other non-antibiotic antimicrobials, such as antivirals, antifungals, and disinfectants, which “*might themselves be entry points for being selective of antibiotic [resistance] problems*” (Day 2 Workshop), noting that looking at antibiotics in isolation would lead to “*proposals that actually do not solve the long-term challenge [of AMR]*” (Day 2 Workshop). As a result of exploring each of the two promising interventions (*taxation of antimicrobials at point of sale* and *infection prevention measures*) through these lenses, participants described three alternative futures: (1) “Tax Burn Out,” (2) “Addressing the Basics,” and (3) “Siloed Nations” (Tables 1, 2). The third scenario emerged from discussions of the “Tax Burn Out” and “Addressing the Basics” scenario. The following describes each future world, followed by which actions participants identified to be necessary starting in 2030 to tackle AMR by 2050 regardless of which alternative future arises. Findings from workshop and interview discussions are presented together where similarities in responses exist, while unique contributions from interview discussions are identified.

**Table 1:** Alternative Futures of AMR in Sweden in 2050 under a changing climate: Effectiveness, and pros and cons.

		**Alternative futures**	
	**Tax burn out** *Participants explored how and why taxation of antimicrobials at point of sale was successful in combatting AMR in Sweden in 2050 under a changing climate*.	**Addressing the basics** *Participants explored how and why infection prevention measures were successful in combatting AMR in Sweden in 2050 under a changing climate*.	**Siloed Nations** *This alternative future emerged from participants' main task, which was to explore alternative worlds where taxation of antimicrobials and infection prevention interventions were each successful in combatting AMR in Sweden in 2050 under a changing climate*.
Effectiveness and pros and cons	Participants did not view taxing antimicrobials at point of sale to be a priority or stand-alone intervention to combat AMR under a changing climate.	Participants viewed infection prevention measures to be a high impact and priority intervention to tackle AMR under a changing climate.	Participants described “Siloed Nations” as a world where nationalism and protectionism prevail and threaten to derail the ideal “Addressing the Basics” future scenario, rendering it an ineffective context to sustainably tackle AMR circa 2050 globally under a changing climate.
	Pros: • Taxing antimicrobials could generate needed awareness about AMR among the public and at policy tables. • Antimicrobial tax income can be used to fund effective multi-pronged interventions that prevent and reduce AMU and the development and spread of AMR.	Pros: • Infection prevention measures will have positive impacts on the prevention of disease and reduced need for AMU in human and agricultural sectors. • Infection prevention measures contribute to the attainment of Sustainable Development Goals (SDGs). Continued implementation of Infection prevention measures globally and the attainment of SDGs will successfully contain AMR to lowest possible levels in Sweden and globally under a changing climate.	Pros: • None discussed by participants.
	Cons: • Taxation of antimicrobials can perpetuate inequities in access to antimicrobials that can save lives.	Cons: • Achieving the SDGs in the year 2050 in Sweden is possible but is questionable at a global level due to insufficient progress in achieving 2030 SDG milestones and because key goals, such as ending poverty and hunger, have been further compromised due to the COVID-19 pandemic.	Cons: • Countries move strategic industries away from global value chains to local value chains, particularly in terms of food, pharmaceutical, and energy production supply. This in turn creates walls between nations that conflict with international cooperation and international trade. Consequently, developed countries' sharing of resources and technologies to build capacity and infrastructure in countries that require the help (namely, low-middle income countries) to address the SDGs and deal with AMR and climatic changes is threatened. The “Addressing the Basics” scenario is potentially derailed. • Siloed Nations catalyzes mass migration of humans and increases the potential for conflicts due to water scarcity. • Increased technological advancements (e.g., robots) to carry out jobs that immigrants commonly fill (e.g., cleaning infrastructure, farming) raises questions about what social safety net and other mechanisms need to be implemented to minimize labor disruptions and associated negative socio-economic wellbeing that can limit people's ability to integrate and contribute to society, particularly in mass migration scenarios.

**Table 2:** Key features of the year 2050 to ensure intervention success in addressing AMR under a changing climate by alternative future.

		**Alternative future scenarios**	
	**Tax burn out** *Participants explored how and why taxation of antimicrobials at point of sale was successful in combatting AMR in Sweden in 2050 under a changing climate*.	**Addressing the basics** *Participants explored how and why infection prevention measures were successful in combatting AMR in Sweden in 2050 under a changing climate*.	**Siloed Nations** *This alternative future emerged from participants' main task, which was to explore alternative worlds where taxation of antimicrobials and infection prevention interventions were each successful in combatting AMR in Sweden in 2050 under a changing climate*.
Key features of the year 2050	Sweden and the world need to look like the “Addressing the Basics” future scenario	***Six key features:*** 1. ***Transformation in collective consciousness*** - Greater economic value placed on health and wellbeing (as opposed to corporate profit). This drives how services are commissioned, suppliers are selected, industries are selected, and what policies prevail. 2. ***Global power and collaboration*** - Global power shifts to the East, which brings forth stronger international cooperation, international codes and enforcement, and common rules that uphold society's redefined values of health and wellbeing. - There is collaboration between countries, and the global sharing of lessons learned and resources (e.g., human capital, technology) that help all countries to achieve the SDGs, and tackle AMR under a changing climate. 3. ***A transformation in social norms and behavior*** - Because the public has seen the impacts of AMR and climate change and are well educated on the issues, society values health and wellbeing and thus pay more for high animal-welfare and sustainable products (e.g., food), use antimicrobials appropriately, eat healthier, are more active, and use new technologies (e.g., wearable devices) that monitor their health. 4. ***Changes in food demands*** - The envisioned global power shift to the East will change which foods are being consumed in the West. - Given greater concerns about the environment and health and wellbeing of humans and animals, greater demand for foods produced sustainably, with high animal welfare standards, appropriate or no antimicrobial use, and no chemicals will be high. - The demand for beef will be lower and cost of beef will grow beyond inflation. - Food production systems will shift to a mix of de-intensification and a few consolidated food production companies that feed the world. - Data modeling will inform what types of food animals to raise based on need for AMU and climate change impacts. 5. ***Innovation and technological developments*** - Food: Breeding animals and crops that are resistant to infections, pests and diseases, genetically modified foods, and CRISPR will reduce the need for AMU and allow foods to be produced in water-scarce conditions. Lab-produced foods (e.g., alternative meats) that eliminate AMU and are nutritious and thus reduce risk factors for disease (e.g., cholesterol) will help contribute to healthier populations, although cultural acceptability may vary. - AMR Research: Discoveries that address bacterial infections will have been made and tackling viral, fungal, and parasitic infections to prevent and control resistance is a key focus. - Smarter AMU is prevalent due to the development of rapid diagnostic testing and markers of infection, and dosing of AMU appropriate to the stage of infection of people and animals in their life cycle. This has reduced AMU metaphylaxis and prescribing practices are improved. - Antimicrobial alternatives, greater understanding of the microbiome and microbiota, and new ways to combat AMR transmission. - Environmental Technological Innovations: Artificial intelligence, Smart cities and societies, circular economies (e.g., to repurpose water and waste) to ensure adequate water and energy supply and proper infrastructure, and traceability systems to identify infectious outbreaks in the food chain will be widespread, helping to contend with climatic changes. 6. ***Migration*** - International collaboration and sharing of resources and countries achieving the SDGs that in turn help drive down inequities help stabilize migration patterns, particularly in low- and middle-income countries, as they would be better equipped to handle AMR and climatic changes in 2050. - Migration will still occur but not on a mass scale. Sweden, which can produce food and has sufficient water supply despite climate change will open its borders to migrants and provide supports needed for their effective settlement and integration into Swedish society.	None discussed by participants.

### Scenario 1: “Tax Burn Out”

In this scenario, participants explored how and why the *taxation of antimicrobials at point of sale* intervention was successful in addressing AMR in Sweden circa 2050 under a changing climate. However, taxation of antimicrobials was not deemed to be a high priority or stand-alone measure by participants. Participants acknowledged that taxation of antimicrobials at point of sale could bring needed attention to AMR at policy tables and among the public. They also acknowledged that the intervention could potentially reduce AMU in food production and humans, providing the intervention is implemented today and is part of a progressive tax system that is refined over the next 30 years by using data science to track behaviors to make this “*blunt instrument…more precise in how it is affecting our behaviors*” (Day 1 workshop). To illustrate, participants noted taxes and tariffs could be increased responsively on different groups of antibiotics that demonstrate increasing resistance based on data, potentially altering the types of antibiotics prescribed and used. Using taxes to incentivize rather than penalize behaviors (e.g., providing rebates to farmers and industries or to humans for using antibiotics in accordance with guidelines and exactly as prescribed) was also viewed to potentially motivate people to use and dispose antimicrobials properly.

However, challenges with imposing a tax were viewed to override the potential benefits of this approach. First, participants noted that high taxes would be needed to evoke desired behavior change in wealthier countries like Sweden, where citizens have drug coverage and broad-spectrum antibiotics are inexpensive to buy, e.g., “*ciprofloxacin is the same price as a box of candy”* (Day 2 workshop). Second, participants stressed that applying taxes to antimicrobials “*can be quite discriminatory on the poor sectors in our community and…the poorer countries in the world to have access to antibiotics that may be lifesaving*” (Day 2 workshop). While taxing those who can afford it and providing subsidies to ensure access for those who are less well off could offset inequities, participants strongly felt taxation would not address the issues that underpin AMR and many public health problems, such as access to nutritious and affordable foods and clean water, proper toilets and proper infrastructure such as housing and waste and wastewater treatment facilities. Third, participants described how different taxation schemes across countries could create inconsistencies and lead to “…*informal markets that bring additional problems…beyond what we have to deal with initially*” (Day 1 workshop). Fourth, building stakeholder buy-in for yet another tax was said to be a hard sell. Finally, participants stressed that if the taxation intervention were to contribute to addressing AMR in 2050, its main contribution would be via the use of tax dollars to fund the types of multipronged interventions that are actually essential to tackling AMR (e.g., developing rapid diagnostics, alternatives to antimicrobials, measures that promote healthy lifestyles and prevent infectious and non-communicable chronic diseases, ensuring access to food, clean water and appropriate housing, employment, and interventions to improve human behavior) and that to be successful these interventions would need to be implemented in a context that reflects the “Addressing the Basics” alternative future scenario described below.

### Scenario 2: “Addressing the Basics”

In this future scenario, we asked participants to explore how and why *infection prevention measures* were successful in addressing AMR in Sweden circa 2050 under a changing climate. Participants identified *infection prevention measures* to be a high impact and essential intervention necessary to address AMR. Participants envisioned that AMR will be effectively contained at low levels in 2050 should current trends of increasing global awareness in human and animal sectors about AMR and growing implementation of infection prevention measures (e.g., on-farm biosecurity, hand hygiene due to the COVID-19 pandemic) continue, and if there is greater focus on ensuring other measures, such as selective animal breeding, access to nutritious feed and food, and clean water and proper toilets become common place. Participants noted that these measures may create “*many positive spillover effects*” (Day 2 workshop) that prevent and control diseases and reduce the need for AMU. Prevention measures were also viewed as critical because they contributed to the achievement of the Sustainable Development Goals (SDGs), and the SDGs were identified to be key to addressing the driving factors that underpin AMR, climate change and other complex public health problems, “*It doesn't really matter what nice drugs we…come up with or…taxation programs and so on…if there is no access to clean water and healthy nutritious food across the globe and hygiene, sanitation…which also [takes] us back to the Sustainable Development Goals…really the basics…we are lost*” (Day 2 workshop).

Participants stressed that the SDGs enable “*reducing inequalities as far as possible because that reduces other risks that can then have implications for AMR, so fuel, poverty, food poverty.”* (Interviewee A), and by achieving the SDGs, participants asserted countries could become economically viable and self-sufficient and better able to address the negative impacts of AMR and climate change. Predicting that human beings will have the same basic needs as today, such as access to clean water, nutritious food, education, employment, and more, participants described six key features of a 2050 future world where the SDGs could be achieved and AMR addressed under a changing climate. Participants were explicit these features were key to achieving this future.

#### A Transformation in Collective Consciousness

Because of the COVID-19 pandemic and heightened concern over AMR and climate change, participants said that by 2050, Sweden and many countries beyond had completely redefined their values and how society operates, with less focus on economic profit and a greater economic valuing of health and wellbeing and reducing inequities. Given this orientation, one interviewee imagined that the World Health Organization would serve as an executive agency of the United Nations with greater authority to provide strong leadership and responsibilities to promote health and wellbeing and address AMR and climate change. Citizens were also described as playing an active role in the political arena “…*finding their voice and insisting on change…in the whole global system of how we work together*” (Interview A) leading to an agreed system for accountability where politicians are voted out by their citizens if they do not act appropriately in their running of the country. Policies were also said to align with the redefined values driving society, rooted in sound science, and to transcend the government of the day to ensure previous commitments prevail. These redefined values were also said to drive how services are commissioned, suppliers are selected, how industries are developed and what policies prevail.

#### Global Power and Collaboration

A shift in global power dynamics from the West to the East was envisioned by participants along with the presence of “*stronger international organizations, [international] cooperation, more international codes and enforcement of common rules*” (Day 2 workshop) that uphold the redefined values of health and wellbeing that underpin how society operates. Collaborations between countries that share borders (e.g., Nordic collaborations and Benelux) were also said to thrive in 2050, enabling learning from each other's experiences and sharing the lessons to help the rest of the world. Sweden, still envisioned by participants to be a European Union member in 2050, was said to be a “*leader*” (Day 2 workshop) in tackling AMR, sustainability, climate change, and helping to realize the SDGs because of their effective application of the European Green Deal and the Farm to Fork Strategy; by applying Sweden's successes and lessons, other countries were seen to be better able to address AMR under a changing climate. High-income countries were also described as sharing human capital, resources and technology with countries that needed it, building their capacity in ways that address the SDGs and, consequently, AMR and climate change.

#### A Transformation in Societal Norms and Behaviors

In line with redefined societal values of health and wellbeing and as a result of education and experiencing the impacts of resistance and climate change in 2050, participants prophesized that the public will eat healthier, be more active, pay more for animal-welfare friendly and sustainable products (e.g., food), and use antimicrobials appropriately. Participants also imagined people using new technologies, such as wearable devices that send early warning alerts to the individual if at risk for a heart attack or other unwanted health events. The above changes were also said to have come about via improved scientific understanding of about how to influence behavior change.

#### Food Demands

While modeling data was said to likely help determine which types of food animal species to farm based on reduced need for AMU or reduced carbon emissions in 2050, participants also stressed that which foods are available will be strongly based on “*who's running the food systems in 2050*” (Day 2 workshop). Increased population growth, wealth, and power in the East was described as having a potentially dramatic effect on global food demands that may change dietary patterns in the West. Participants noted these demands could lean toward the East adopting Westernized diets, vegetarian diets, or diets containing far less beef and pork and more poultry and fish. Insect meal was also said to feature to some extent in human diets because of their biomass but may be more likely be used in animal feed. However, given the climate change reality and greater concerns about the environment and animal welfare among the public, participants felt food demands would likely lead to a reliance on de-intensification of food production practices and growth of niche markets that promote animal welfare, use antimicrobials appropriately, and reduce the use of chemicals. Participants also noted that beef prices would “*increase beyond inflation*” (Day 2 workshop) and be consumed primarily on special occasions. Another view was that both de-intensification and a few large and regulated food production companies would exist to feed the world, and that fewer food animal species would be produced. The potential of producing fewer species was viewed “*a double-edged sword*,” (Day 2 workshop) as reducing biodiversity could increase the risk of disease emergence that can more easily wipe out the food supply but could also enable greater standardization in farming practices, such as appropriate AMU and more people working on the same species globally so that when problems arise, they can be solved efficiently as a priority.

#### Innovation and Technological Developments

Regardless of the food production systems that prevail, participants stressed that innovation and technological advancements would be essential to ensure the negative consequences of AMR and a changing climate are minimized.

##### Food Innovations

Breeding animals and crops with natural resistance to infections, pests and diseases was identified as a “*very important element to reducing… the need for antibiotics…*” (Day 2 workshop) under a changing climate. Producing foods using genetically modified organisms (GMOs) or Clustered Regularly Interspaced Short Palindromic Repeat (CRISPR) technology was said to “*take you to places that are impossible*” (Day 2 workshop) at present, as they could help to grow food that could reduce the need for AMU and can thrive in water-scarce conditions. Lab-produced foods (e.g., alternative meats) that do not require AMU, are nutritious, and help to reduce health problems such as cholesterol that in turn could contribute to healthier populations, were also envisioned to be more prominent in 2050, with differing opinions on their widespread cultural acceptability. Making food production “*too artificial*,” (Day 1 workshop) however, was said to potentially limit animal manure use on crops, which may shift the system to rely on artificial manure that could limit soil nutrients or rely on human waste that necessitate waste management systems that are effective in clearing, for instance, antimicrobial residues. Changes in food production were cautioned to potentially lead to job losses for people, such as if food production hinges on a few large-scale corporations.

##### Research on AMR

Participants noted that by 2050, significant discoveries that address bacterial infections will have been made and progress toward tackling viral, fungal, and parasitic infections to prevent and control resistance made. “*Smarter use of antimicrobials*” (Day 1 workshop) was also described to be a reality in 2050 via the development of early and rapid diagnostic testing and markers of infection and resistant organisms that make dosing “…*optimal, precise and responsive to the stage of infection*” (Day 1 workshop) in people and animals, ultimately reducing on-farm AMU for metaphylaxis and improving prescribing practices. Antimicrobial alternatives (e.g., new feeding strategies, feed additives, new vaccines), and greater understanding of the microbiome and microbiota and new ways to addressing AMR transmission that help to reduce contamination in land and water courses in both humans and animals were also described to exist and be widely used in 2050.

##### Environmental Technological Innovations

While achieving the SDGs was emphasized to enable countries to become self-sufficient and better able to manage the negative impacts of climate change and AMR, climate-induced water scarcity was said to likely make it “*tougher*” (Day 2 workshop) to achieve the basics particularly in low- and middle-income countries. Thus, new developments such as desalinization plants in rural communities and greater access to wells and systems that provide clean water for populations were expected to be well established in 2050 in countries that need them. Also anticipated was widespread smart cities and smart societies that use data to ensure core infrastructure elements are providing adequate clean water and electricity supply; proper sanitation, including waste and wastewater management; and circular economies that repurpose existing waste in ways that support development efforts and minimize inequity gaps globally (e.g., using treated human waste as a source for nutrients in agriculture to address food insecurity). One interviewee imagined a traceability system that provides a holistic look over the food supply chain, detecting infectious pathogen emergence at its source and helping to ensure a safe and secure food supply. Artificial intelligence (AI) enabling the processing of information akin to the human brain was said to take rapid diagnostics further by being able to pinpoint infectious disease outbreaks and other safety and prevention issues before they happen at a societal level to facilitate better macro-level decision-making about how best to intervene.

#### Migration

Population growth, poor infrastructure and social support systems, and water scarcity in low- and middle-income countries, and large land masses being submerged underwater in historically food-producing countries were said to have potential to drive mass migration, conflicts, and wars. However, international collaboration and the less varied inequities globally due to Sweden and other nations' achievement of the SDGs were envisioned to stabilize migration patterns because countries, particularly in low- and middle-income regions, would be better able to handle AMR and climatic changes in 2050. Sweden was also envisioned to help those who do migrate: participants noted that Sweden's aging population and stagnant population growth, commitment to addressing inequities, and ability to produce sufficient food due to longer harvest periods and sufficient water supply in certain areas as described in the news story of Sweden in 2050 under climate change (36) used in our study, would drive Sweden to take responsibility to produce more food and open its borders to migrants. One interviewee envisioned that immigration policies in Sweden would change to ensure effective integration into society and uphold success with tackling AMR under climate change conditions. These policies would involve screening procedures that admit newcomers with desirable skills and who share the same collective values of health and wellbeing that govern the country. Sweden would also invest heavily into supporting immigrants to learn the Swedish language and provide access to education and employment opportunities to facilitate their active participation in Swedish life.

### Scenario 3: Siloed Nations

The “Siloed Nations” alternative future emerged as a result of participant discussions of the “Tax Burn Out” and the ideal “Addressing the Basics” scenarios. Here, participants described an alternative future where nationalism and protectionism prevail and threaten to derail or destabilize the ideal “Addressing the Basics” future. Participants described the emergence of populistic politicians coupled with the growing trend away from globalization toward nationalism over the past 30–40 years that intensified with the COVID-19 pandemic, to have led some national governments to reassess strategic industries, particularly the pharmaceutical, food and energy sectors, and move them “*away from the more efficient global chains to local value chains with a view to ensuring security of [domestic] supply*” (Day 2 workshop). One interviewee imagined Sweden and many other countries as becoming independent in, for example, the energy sector to serve the dual purpose of contending with climate change while protecting national interests and avoiding conflicts that could arise when other countries are relied upon for energy needs or other supplies. However, these efforts to “*build a wall…*” (Day 2 workshop) were said to directly conflict with international cooperation and international trade, which in turn was viewed to threaten the global sharing of resources and technology to build capacity and infrastructure in countries that require the help, particularly developing countries, to deal with AMR and climatic changes. These conditions were said to create instability and catalyze mass migration and increase the potential for conflicts due to water scarcity. Interviewees also described how technological advancements, such as robots and increased automation to carry out jobs that immigrants often fill including working in the farming and service industries and cleaning of infrastructure (e.g., subways), were also said to raise question about what social safety net and other mechanisms need to be instituted to minimize labor market disruptions and associated negative socio-economic wellbeing that can limit people's ability to integrate and contribute to society, particularly in mass migration scenarios.

### Necessary Actions for 2050 Success

**Figure 2:**
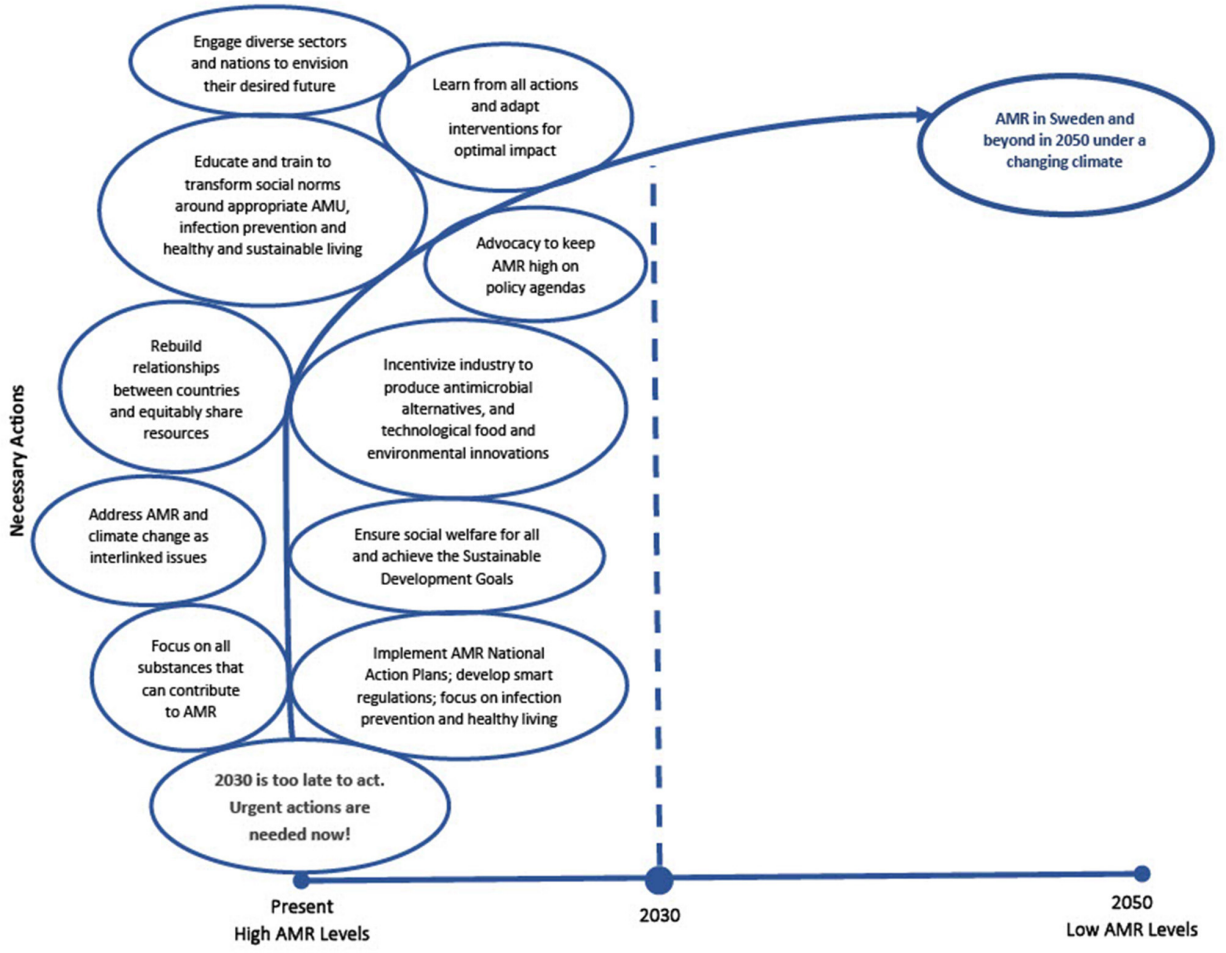
Actions to take in 2030 to address AMR in 2050.

After exploring the future, we had participants travel to the year 2030, to describe the actions that need to be taken starting at that timeframe to ensure AMR interventions are successful in 2050 under a changing climate, regardless of which future scenario unfolds. Participants clearly stated that AMR is an urgent crisis and if we wait until 2030 to act, we will be too late in being able to tackle it. Participants stressed that “*we need to…move now*” (Day 1 workshop) and identified the actions outlined below and illustrated in Figure 2 that must be taken immediately.

#### Make AMR “Sexy”

Re-establishing AMR as an urgent problem regardless of new crises was identified to be critical. But AMR was described as “*not sexy enough*” (Day 1 workshop) because leaders are more interested in exploring new frontiers such as via space exploration. Also, the COVID-19 pandemic, which has eroded international collaborations, created an “*economic disaster*” (Day 1 workshop), increased mental health issues, and generated a mistrust in science, has led people to switch off hearing about any further crisis. However, participants believed these conditions offer ripe opportunities to “*highlight how another bigger pandemic can be coming down the pipeline, which would be AMR*” (Day 1 workshop) and to use the lack of preparedness of nations to deal with the COVID-19 pandemic to convince governments and the public to invest in long-term planning, and ultimately move AMR up the policy agenda. Advocacy was identified as the vehicle to mobilize these messages to land and keep AMR high on the policy agenda even when new crises arise and despite the long time it takes for decision-makers to see the positive gains of their investments.

#### Take a Holistic Approach

Taking a holistic approach characterized by coordinated and multi-pronged actions that directly or indirectly impact AMU or AMR across the One Health system at a global scale were identified as necessary. Expanding the focus to include all agents that contribute to resistance (not solely antibiotic use) and implementing AMR National Action Plans in all countries were deemed to be imperative. Developing smart regulations that involve diverse stakeholders in the design of regulatory standards to address unintended consequences to different actors from the outset was also noted as important by participants. Improving surveillance and monitoring of AMU by gathering unified data globally on actual AMU and antimicrobial disposal was identified to be important given advancements in precision medicine which may erroneously show AMU reducing even though drug potency is increasing. Continued efforts to develop rapid diagnostics, understand animal species-specific dosing of antimicrobials appropriate for the infection and stage of species lifecycle, and research on the microbiome and development of alternatives to antimicrobials were additional measures that participants discussed.

A greater focus on promoting healthy lifestyles and preventing illness in humans and animals coupled with de-intensification of food production practices were viewed to be important to tackling AMR. Participants also noted a need to implement effective infection prevention and control measures at airports and to institute a third-party body to assess and improve measures to minimize infectious illness and AMR spread via international travel. One interviewee suggested developing AMR screenings for travelers, immigrants, and as part of health care interventions that require blood pressure and full blood tests as additional measures to avoid reintroducing pathogens eliminated in a given nation or to determine prescribing practices.

Because of the importance of innovation to tackle AMR under a changing climate, participants stressed a need to find attractive investment strategies to incentivize industry interest and commitment to develop antibiotics and alternatives such as new feeding strategies, feed additives, bacteriophages, vaccines, probiotics, and provide aid funds to companies for technological and infrastructure development to address AMR and climate change. Many of the technologies described in 2050 were said to already exist now but require further development and participants stressed a need for transparent discussions regarding unintended consequences that may arise should technologies, such as GMOs and AI, become widely adopted. Determining how to deal with potential job losses that may result from technological advancements that enable movement away from use of fossil fuels or changes in agricultural food production systems was also identified as needed.

#### Couple Up AMR and Climate Change

Participants stressed the importance of keeping “*the climate change discussion and AMR, two global issues, closely linked together because there's lots of interactions between both*” (Day 2 workshop). Greenhouse gas emissions were described to be already driving de-intensification in some profitable sectors within some countries with intensive high-stocking agriculture. Growing consideration of environmental costs of current actions in turn were said to continue to impact how food is produced, such as a greater orientation to sustainable practices that could have implications for AMU and efforts to prevent AMR development and spread.

#### Achieve the Sustainable Development Goals

Achieving the SDGs was identified as key to addressing AMR under a changing climate in 2050. Most participants questioned whether this was feasible due to insufficient progress in achieving 2030 milestones and because key goals, such as ending poverty and hunger, have been further compromised due to the COVID-19 pandemic. Creating employment opportunities and achieving “*a certain level of welfare for all the population*” (Day 1 workshop) were two key elements participants described as necessary to recover from the COVID-19 global economic impact, increase people's ability to make better choices (e.g., willingness to pay more for food to enable appropriate AMU and sustainable practices in food production), and get back on track to realize the SDGs. Exploring animal welfare as an under-examined pathway toward achieving the SDGs was also identified.

#### Rebuild Bridges

“*Rebuilding those bridges that were broken in 2020 due to the pandemic*” (Day 1 workshop) was identified a priority to realize the SDGs and address AMR and climate change. Fostering international cooperation to reverse “*nationalism…and the division between countries*” (Day 2 workshop) that have led to budget cuts that compromise human rights and the achievement of the SDGs was noted. Educating countries and farmers about the future economic impacts of AMR and how infection prevention measures can positively impact economic prosperity was said to “*take you a long way toward solving AMR without ever needing to talk about it… [make it] easier to get buy-in from key stakeholders*,” (Day 2 workshop), and allows learning from experiences around a shared interest across sectors globally which helps foster collaboration. Developed countries taking responsibility to solve problems they cause in other parts of the world was another leverage point identified to rebuild relationships. Eliminating AMU in food production in Sweden, for instance, while sourcing food from developing countries that use antimicrobials to meet market demands was said to shift the problem elsewhere. One suggested action was to test for AMR in imported foods as one opportunity of how to put pressure on exporting countries to improve their production practices; this was also seen as a way for developed importing countries to share resources and help to build infrastructure in exporting countries that need it, to enable them to remain economically viable in the marketplace, which will better prepare them to curb the negative impacts of AMR under a changing climate.

#### Transform Societal Norms

Participants stressed that long-term societal and cross-sector commitment and changes in norms and behaviors are imperative to realize the SDGs, and address AMR under a changing climate. Thus, engaging the public was deemed important. Also important were strategies such as identifying policy levers that tie AMR and climate change to food prices and insurance policies to make the issue relevant to individuals' everyday lives, labeling the antibiotic and carbon footprint of food products using a unified metric to inform purchasing decisions, and using testimonial-based communication campaigns featuring hard-hitting messages about living with the negative impacts of AMR and how to use antimicrobials appropriately to motivate behavior change. Educating students throughout their educational trajectory about AMR, infection prevention, animal welfare, sustainability, healthy eating, physical activity, public health, and how humans connect to ecological systems, was identified as important to improving healthy lifestyles, reducing disease and AMU, and protecting the environment. Cross sector education and training on the benefits and harms of antimicrobials, which antimicrobials to use when and how to use appropriately, the interlinkages between AMR and climate change, and infection prevention measures were deemed to be essential, particularly among farmers who use antimicrobials, and among prescribers, dispensers and retailers who determine which drugs or products are sold and used. Engaging social scientists to help develop interventions that can change behaviors and overcome people's desire for “*quick fixes*” (Day 1 workshop) were also identified.

#### Learn From Actions

Implementing, surveilling, monitoring and evaluating AMR National Action Plans, climate change policies, and interventions to address the SDGs were identified as necessary to identify best practices, demonstrate progress, advocate for continued investment, and adapt actions in response to changing circumstances to garner policy makers' commitment and keep these issues high on policy agendas. Documenting and sharing best practices and lessons learned from countries that have demonstrated social and economic success in, for instance, reducing antibiotic use in food animals, improving animal welfare standards, or achieving a SDG goal, were deemed to be an important strategy for others to learn how to apply and scale similar practices in their own jurisdictions. The COVID-19 pandemic was also identified to offer a wealth of knowledge that can inform and improve actions to address AMR under a changing climate by demonstrating the importance of infection prevention measures and how “*we can reduce the amount of spreading diseases…by ourselves by washing hands, keeping distance and so on*” (Day 2 workshop); illustrating the negative consequences of poor international collaboration; devising strategies to counter politicians from politicizing evidence for their political agendas; creating consistent communications about evidence to not undermine confidence in scientific experts; improving risk mapping to better prepare for future pandemics like AMR; and showing how to influence behavior change via “*nudging*” (Day 1 workshop).

#### Envision the Future

Interviewees identified a need for ongoing vision development where different nations and sectors construct their ideal future scenarios of the world in 2050 and use these visions to find commonalities and ideas about how to address problems like AMR effectively, sustainably, and ethically. Also key was for emerging global powers to learn from the successes and mistakes of their predecessors to create a better vision of the future and how they want their actions to impact themselves and the collective, such as the value they place on producing food that is healthy for consumption and for the environment.

## Discussion

We applied scenario planning methods to explore how a high-income country (Sweden) might address AMR under a changing climate in 2050, a method that has been marked as useful to better tackle complex public health problems (28). Specifically, we used a participatory approach involving experts from multiple sectors that are both traditionally and less traditionally engaged in AMR discussions. By exploring each of two promising interventions (*taxation of antimicrobials at point of sale* and *infection prevention measures*), participants constructed three alternative futures, contemplated their benefits and consequences, and identified necessary actions for long-term AMR mitigation success.

Strategic priorities (13) and knowledge of promising actions (29) to address AMR exist, and scenario planning has also been previously applied. Harbarth and Samore (38) foretell an ideal 2025 future of AMR with a focus on humans and healthcare where: policies and behavior change interventions optimize prescribing habits and social norms around appropriate AMU in humans; technological advancements map the human microbiome and lead to new infection prevention probiotic therapies; greater data sharing and international cooperation enables consistent application of infection control and public health policies that decrease over-use of antimicrobials; and new antimicrobials and appropriate use of existing antimicrobials render deaths from pan-resistant infections with no treatment rare. More recently, García et al. (39) identified four alternative futures of AMR in 2050 based on AMR progress and varied levels of support by the European Commission, and highlighted a need for increased coordination and commitment in Europe and internationally, increased mass-reach campaigns to raise public awareness of the AMR threat, the development of new diagnostics and antimicrobials and better prescribing practices, and efforts to curb AMR transmission in the environment and via travel. Our study shares some similarities with these studies and expands existing thinking in four ways. First, it brings a climate change lens to the AMR issue and within this context it considers a wider system that extends beyond Sweden to the global scale and encompasses key drivers (e.g., societal values, governance structures, economic growth, consumer spending, food production and environmental technological advancements) that could impact future efforts to address AMR as climate change intensifies. Second, it identifies *infection prevention* measures as an essential intervention to mitigate AMR long-term under a changing climate because of cross-sector relevance, scalability, and contributions to achieving the SDGs. It also identifies *taxation of antimicrobials at point of sale* to be a low-impact intervention that could generate tax income to fund effective multipronged interventions but would exacerbate inequities that drive AMR. Third, it paints an ideal alternative future that stresses the achievement of the SDGs to be required for the success of any intervention to tackle AMR under a changing climate, and describes a less ideal future where nationalism compromises necessary global cooperation, thereby unraveling efforts to achieve the SDGs. Fourth, our study applied a participatory approach and through participating in the workshops or interviews, participants were able to identify a critical need for different sectors and levels of the system to take specific actions to address AMR without delay. Key to success is a holistic approach involving advocacy, implementation of National Action Plans, addressing AMR and climate change as interlinked issues, and improving social welfare as an important step toward addressing the SDGs. The holistic approach hinges on rebuilding international collaboration, collectively adapting actions based on evaluations and lessons learned, and ensuring continued scenario planning exercises involving stakeholders from human, animal and environmental sectors and high-income and low- and middle-income nations to envision and work toward desired futures.

### Strengths and Limitations

Applying the scenario planning approach led to the creation of three alternative futures that made transparent the complexities that underpin AMR mitigation, and identified several conditions and actions for policy actors' (researchers, practitioners, and policy makers) considerations when planning how to address AMR under a changing climate. Our participatory approach was key to generating this knowledge. By bringing together experts that are traditionally and less traditionally engaged in discussions about AMR from human, animal and agricultural sectors, participants were able to share and build on each other's diverse areas of expertise and ideas, and question each other's assumptions, to construct potential pathways to AMR success in 2050.

Three criteria to assess the quality of scenarios exist: (1) relevance, in that the scenarios address concerns relevant to users; (2) challenging, in that the scenarios get stakeholders to think about the focal problem differently; and (3) plausible, in that the assumptions and events described are internally consistent (e.g., identified events are compatible with the 2050 timeline), researched, and possible (40). Through a feedback process, our participants requested permission to bring the innovative ideas in the scenarios for discussion with their own professional networks to advocate for particular actions, suggesting that the scenarios address specific concerns relevant to their work and are both plausible and stretched their thinking about how they might address AMR differently than their current approach. We also ensured plausibility by grounding the climate change conditions that formed the basis of our mock newsletter (36) in the scientific literature. Further, the interventions selected for exploration exist and are currently operationalized in varied ways (41, 42). Finally, our expert participants drew on professional experiences, trends, and evidence from their respective fields to construct each scenario, and through questioning assumptions, participants worked toward creating consistency in the events described therein contributing to the plausibility of our scenarios.

Although we successfully recruited participants representing diverse perspectives, the perspectives were not exhaustive, and the inclusion of experts from other disciplines may have introduced additional elements to the future scenarios. Future efforts would benefit to institute an iterative process involving other research, practice, and policy actors to further assess if and how these scenarios are relevant, challenging, and plausible to others beyond those engaged in this present study.

#### Implications

Our study demonstrates the value of exploring alternative futures because it provides a way to identify potential risk factors and risk mitigation actions to realize a desired goal regardless which future unfolds. By constructing three alternative futures, participants were able to identify several actions that strive to take us to an ideal world where AMR is addressed under a changing climate circa 2050. These actions (e.g., achieving the SDGs) aim to address the systemic drivers of AMU and AMR, implicate different government departments and sectors, and thus suggest that an all-of-society and whole-of-government approach is needed to address AMR long-term under climate change conditions.

Given the identified need to continue to protect the efficacy of antimicrobials, for AMR and climate change to be addressed as interlinked issues, for the SDGs to be achieved, and for societal norms to change, future research is needed to elicit different stakeholder perspectives including from pharmaceutical, climate change and environment, energy and information technology, economic and insurance, tourism, immigration, and food and farming sectors, as well as social policy actors, advocacy groups (e.g., consumer), and the public. Since our study's focus on Sweden reflects a social welfare state with high levels of confidence in core national institutions and political trust (43) and where awareness about AMR is high among the general population and government and industry actions have reduced AMR (44), engaging stakeholders from countries with different contexts will be important, such as South East Asia where AMR emergence and spread is a growing problem (45). Our scenario planning approach and tools (newsletter, video, and narrative of alternative future scenarios) can be used to engage such stakeholders to assess scenario quality and apply them in planning and decision-making processes by testing how potential actions might hold up under each scenario and identify additional potential risks and mitigation actions to address AMR under a changing climate. Moreover, stakeholders could expand the “Siloed Nations” alternative future that resulted from participants' main task of exploring how and why *taxation of antimicrobials at point of sale* and *infection prevention measures* were each successful in addressing AMR in 2050 under a changing climate. Here, stakeholders can explore how the “Siloed Nations” alternative future could address AMR, including the six key features highlighted in the “Addressing the Basics” scenario.

Further work to explain the interconnections between AMR and climate change, and AMR and the SDGs, is important to formulate comprehensive multi-pronged interventions. Efforts can build on existing work that examines AMR and climate change through a social justice lens (22) and the interface between SDGs and AMR (20, 46–48). Achieving many of the SDGs is recognized as conditional on addressing AMR and AMR is specifically referred to in the SDGs with two new additions relevant to the SDG 3 of good health and wellbeing, including SDG 3.d.2 *(percentage of bloodstream infections due to selected antimicrobial-resistant organisms)*, and SDG 3.d.3 *(proportion of health facilities that have a core set of relevant essential medicines available and affordable on a sustainable basis)* (47). Given participants' anticipation of growing consumer demand for high animal-welfare friendly food products, continued research to explicate the intersections between animal welfare and the SDGs (49, 50) and AMR could be explored.

Identifying interventions that jointly address AMR and climate change, including their benefits and drawbacks in terms of effectively mitigating AMR and keeping AMR high on policy agendas, and interventions that achieve specific SDGs with positive impacts on AMR and vice-versa, will be important to assess. This will require expanding our focus from antibiotics to all substances that drive resistance (e.g., antimicrobials, pesticides, heavy metals), and developing integrated surveillance systems that track the use of these substances, AMR, and climate change indicators across the One Health spectrum. It will also require ongoing assessments about how AMR National Action Plans and other relevant interventions such as infection prevention measures, health promotion and chronic disease prevention interventions, and SDG relevant interventions impact AMU behaviors, AMR, climate change, illness, death, and associated social and economic outcomes.

Because participants described how a shift in global power to the East could impact the West such as food demands and identified a need for greater international cooperation, future research would benefit to explore how high-income and low- to middle-income countries influence one another now and under alternate future scenarios of climate change and power and governance change.

Developing funding mechanisms to incentivize industries to develop technologies and antimicrobial alternatives are needed. Assessing the benefits, long-term consequences, and ethics of technologies (e.g., genetically modified foods in a changing climate) and ensuring transparent discussions involving governments and the public will be important to making decisions about where to direct funding.

Greater attention to engaging and building the capacity of advocates (e.g., NGOs) from human and animal sectors and the media to translate evidence to decision-makers and the public is also necessary to raise and maintain AMR as a high-priority issue on national and international policy agendas.

## Conclusions

Our participatory scenario planning approach enabled participants to create shared visions and identify actions that must be taken now that hinge on fostering cross-sector and global collaboration to address AMR and climate change together, promote health and prevent disease, and achieve the SDGs to effectively address AMR under a changing climate. Taking immediate actions to address AMR will help build resilience toward the changes brought by climate change, and help ensure the provision of food, health, and overall wellbeing over time.

## Data Availability Statement

The raw data supporting the conclusions of this article will be made available by the authors, without undue reservation.

## Ethics Statement

The studies involving human participants were reviewed and approved by University of Waterloo Research Ethics Committe (ORE#41781). The patients/participants provided their written informed consent to participate in this study.

## Author Contributions

SEM conceptualized the study design along with EJP, CAC, DW, and PSJ. IAL, SEM, EJP, CAC, and MC were involved in developing the study methods and data collection materials. IAL, SEM, EJP, CAC, MC, TG, AL, PH, MT, DW, and PSJ contributed to methods and the selection of interventions that were explored in our study. IAL, EJP, MC, APD, RG, BSL, and BM were involved with data gathering. IAL and SA led data analysis and all co-authors contributed. IAL wrote the manuscript. All co-authors read, provided revisions in the form of intellectual content, edits and all co-authors approved the manuscript.

## Funding

This study was funded through an operating grant of the 5th Joint Programming Initiative on Antimicrobial Resistance (JPIAMR 2017). Funding was provided by an operating grant from the Canadian Institutes for Health Research (Institute of Infection and Immunity, Institute of Population and Public Health) (PI: SM, grant number 155210), a Swedish Research Council grant (PI and project consortium coordinator: PSJ, grant number 2017-05981); and an operating grant from the Swiss National Science Foundation (PI: DW, grant number 40AR40_180189). MT and PH acknowledge support from FORMAS (2016-00227). PH was partially funded by FORMAS Inequality and the Biosphere project (2020-00454) and CGIAR Research Programs on Fish Agri-Food Systems (FISH) led by WorldFish. The funders had no role in the study design, analysis, interpretation of data, or the writing of this article or the decision to submit it for publication.

## Conflict of Interest

BM is an employee of the Swedish Pharmaceutical Industry Association, and was previously employed by Pfizer AB. He is also a shareholder of several pharmaceutical companies. The remaining authors declare that the research was conducted in the absence of any commercial or financial relationships that could be construed as a potential conflict of interest.

## Publisher's Note

All claims expressed in this article are solely those of the authors and do not necessarily represent those of their affiliated organizations, or those of the publisher, the editors and the reviewers. Any product that may be evaluated in this article, or claim that may be made by its manufacturer, is not guaranteed or endorsed by the publisher.
